# Supplementary material for: Heritability and interindividual variability of regional structure-function coupling
Source: Nat Commun. 2021 Aug 12;12:4894. doi: 10.1038/s41467-021-25184-4 (PMC8361191; doi:10.1038/s41467-021-25184-4)
Supplement: Supplementary file 1 — Supplementary Information [file 41467_2021_25184_MOESM1_ESM.pdf]

Supplementary Information for manuscript: *Heritability and interindividual variability of regional structure-function coupling*

Zijin Gu,[1] Keith Wakefield Jamison,[2] Mert R. Sabuncu,[1] Amy Kuceyeski [2]*

[1]Department of Electrical and Computer Engineering,
Cornell University, Ithaca, New York, USA
[2]Department of Radiology,
Weill Cornell Medicine, New York, New York, USA

*To whom correspondence should be addressed; E-mail: amk2012@med.cornell.edu.

Supplementary Figures

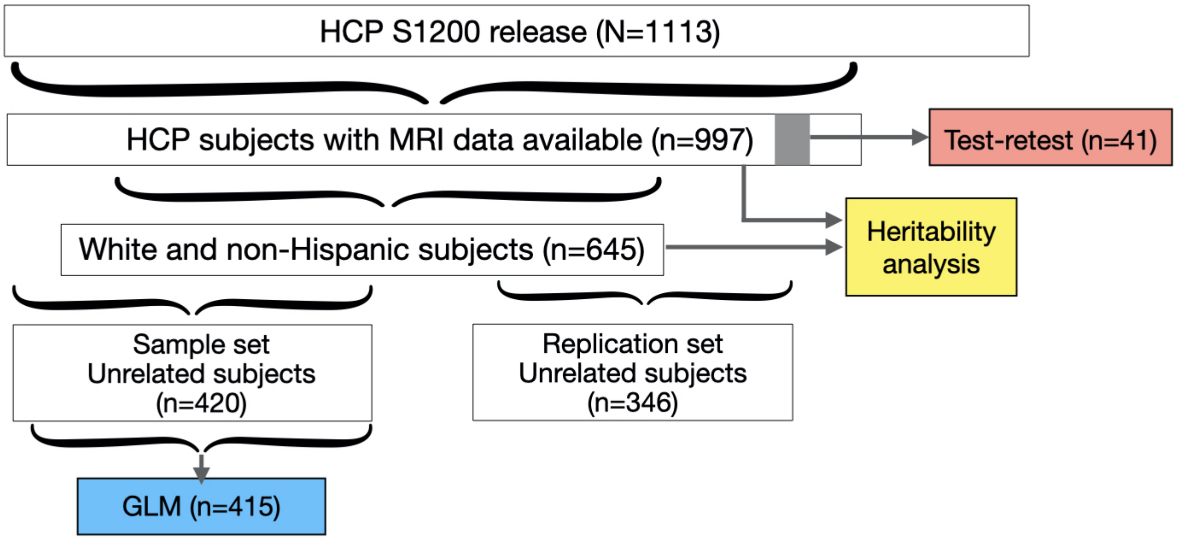

Supplementary Figure 1: Flowchart illustrating the selection of the HCP data for each of the analyses. We began with the final S1200 HCP release, of which only 941 subjects had all four resting-state functional and diffusion MRI scans. We used these 941 in the heritability analyses in the main paper and 645 of these subjects that were white and non-Hispanic in the subgroup analysis included in Figure S5. A set of 41 of the 941 had another visit 6 months after the initial visit, which comprised the group of individuals in the test-retest analysis. An unrelated subset of 420 out of the 941 were randomly chosen for the calculation of the SC-FC coupling; 415 out of this set of 420 had composite cognition scores and were included in the GLM analysis. A second set of 346 unrelated individuals (non-overlapping with the 420) was selected for the out-of-sample validation study.

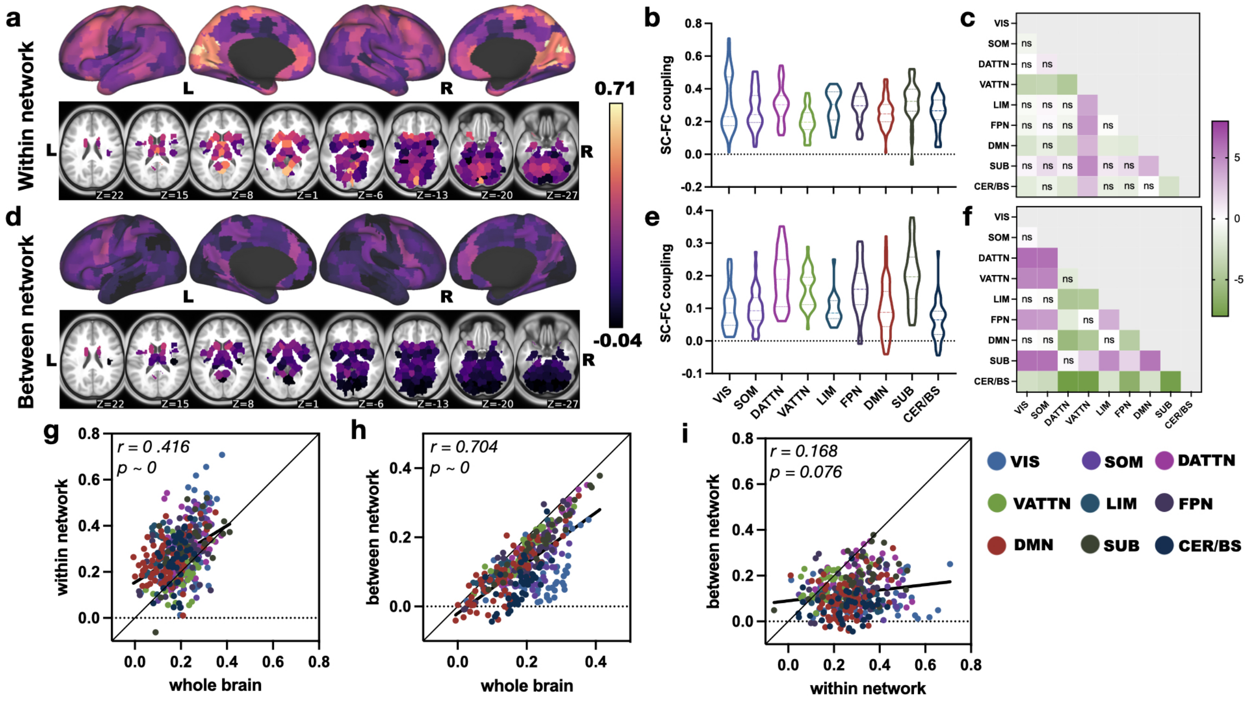

Supplementary Figure 2: Within network and between network SC-FC coupling. **a** Within-network SC-FC coupling for each region is the Spearman correlation of the structural and functional connections between that region and other regions in the same network **b** Within network SC-FC coupling for the nine different networks. **c** shows the t-statistics for all pairwise comparisons of within network SC-FC coupling across networks, calculated as the network on the y-axis versus the network on the x-axis. One-sided p values were calculated (see detailed description in the Methods section). Those comparisons with FDR corrected $p > 0.05$ are marked with ns. **d** Between-network SC-FC coupling for each region is the Spearman correlation of the structural and functional connections between that region and other regions outside of its assigned network. **e** Between network SC-FC coupling in nine different networks. **f** shows the t-statistics for all pairwise comparisons of between network SC-FC coupling across networks, calculated as the network on the y-axis versus the network on the x-axis. One-sided p values were calculated (see detailed description in the Methods section). Those comparisons with FDR corrected $p > 0.05$ are marked with ns. **g** Relationship between whole brain SC-FC coupling and the within-network SC-FC coupling (Pearson's $r = 0.416$, one-sided $p = 0$). **h** Relationship between whole brain SC-FC coupling and the between-network SC-FC coupling (Pearson's $r = 0.704$, one-sided $p = 0$). **i** Relationship between within- and between-network SC-FC coupling (Pearson's $r = 0.168$, one-sided $p = 8e - 4$).

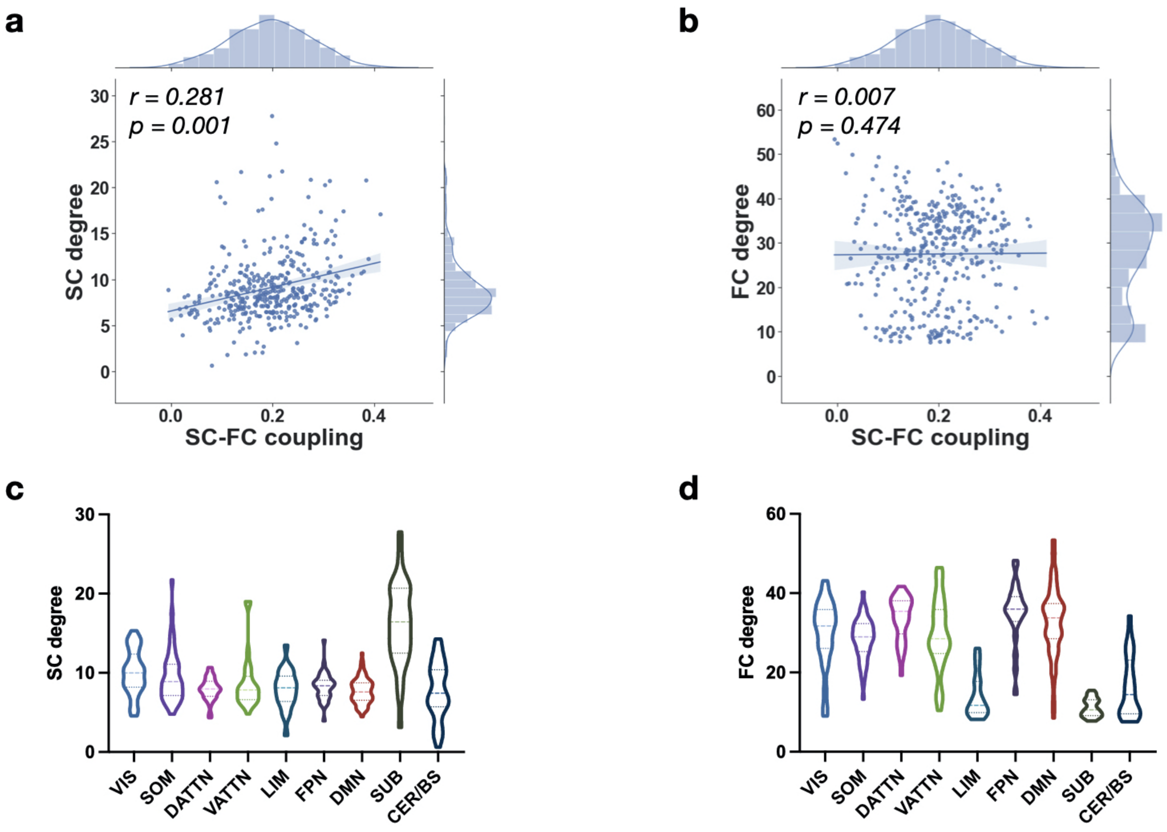

Supplementary Figure 3: Relationship of SC-FC coupling with SC and FC. **a** Scatter plot of SC-FC coupling with the degree of SC. SC-FC coupling has a moderate positive correlation with the degree of SC (Pearson's $r = 0.281$, one-sided $p = 0.001$). **b** Scatter plot of SC-FC coupling with the degree of FC. SC-FC coupling has no significant correlation with the degree of FC (Pearson's $r = 0.007$, one-sided $p = 0.474$). The translucent bands around the regression line represent 95% confidence interval for the regression estimate. **c** SC node degree by network. **d** FC node degree by network.

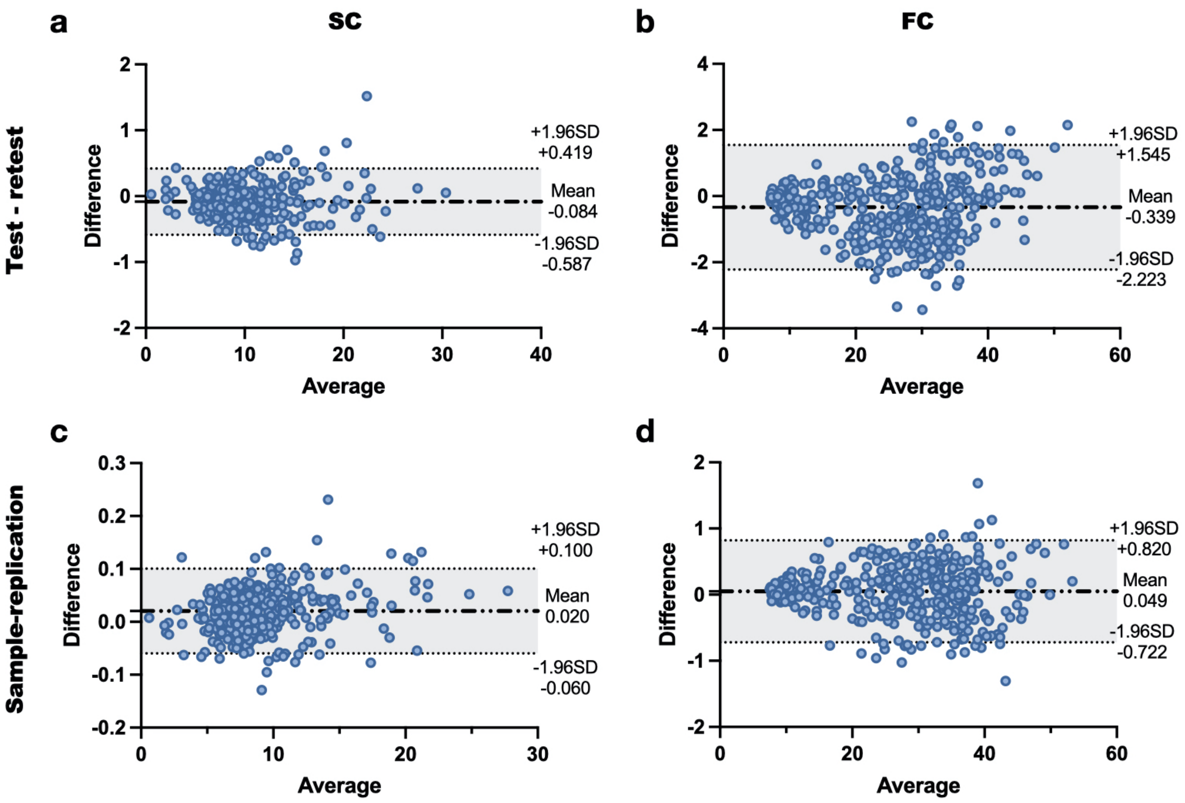

Supplementary Figure 4: Test-retest and out-of-sample reliability of SC and FC node strength. Bland-Altmann plots of the average of the two measures (test/retest or sample/out-of-sample) against the difference in the two measures. We see generally that the two measures are quite reliable across time and across populations (test-retest Pearson correlation for FC and SC was $r = 0.995$ and $r = 0.998$, sample and out-of-sample Pearson correlation for FC and SC was $r = 0.999$ and $r = 0.999$).

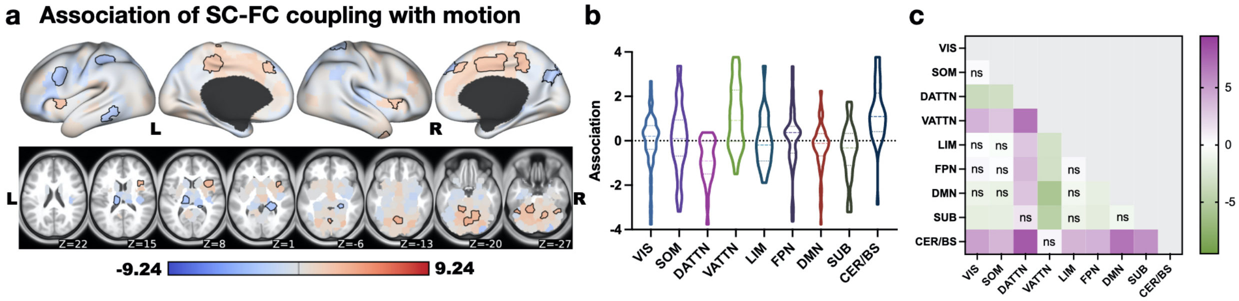

Supplementary Figure 5: Associations between SC-FC coupling and motion. **a** displays regional β values from the GLM quantifying associations between SC-FC coupling and motion. Areas with significant β values (after correction) are outlined in black. **b** shows the network-wise β values for motion. **c** shows the t-statistics for all pairwise comparisons of associations across networks, calculated as the network on the y-axis versus the network on the x-axis. One-sided p values were calculated (see detailed description in the Methods section). Those comparisons with FDR corrected $p > 0.05$ are marked with ns.

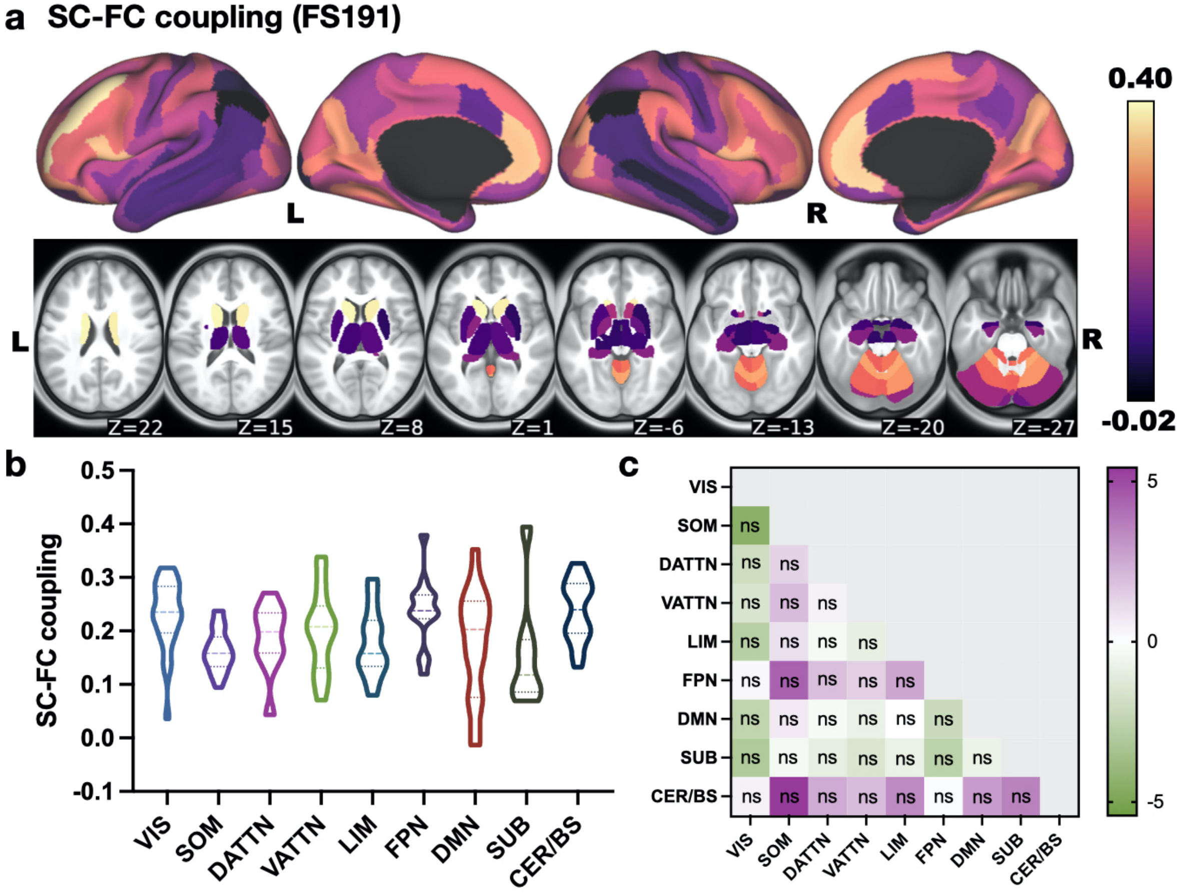

Supplementary Figure 6: SC-FC coupling in FS191 atlas. **a** SC-FC coupling in FS191 atlas varies across cortical and subcortical areas with range -0.02 to 0.40. **b** SC-FC coupling distribution in nine networks. Visual, frontal parietal network, cerebellum and brain stem had generally higher coupling than other areas, with mean coupling 0.23 ± 0.06, 0.24 ± 0.06 and 0.24 ± 0.06, respectively. **c** shows the t-statistics for all pairwise comparisons of SC-FC coupling in FS191 atlas across networks, calculated as the network on the y-axis versus the network on the x-axis. One-sided p values were calculated (see detailed description in the Methods section). Those comparisons with FDR corrected $p > 0.05$ are marked with ns.

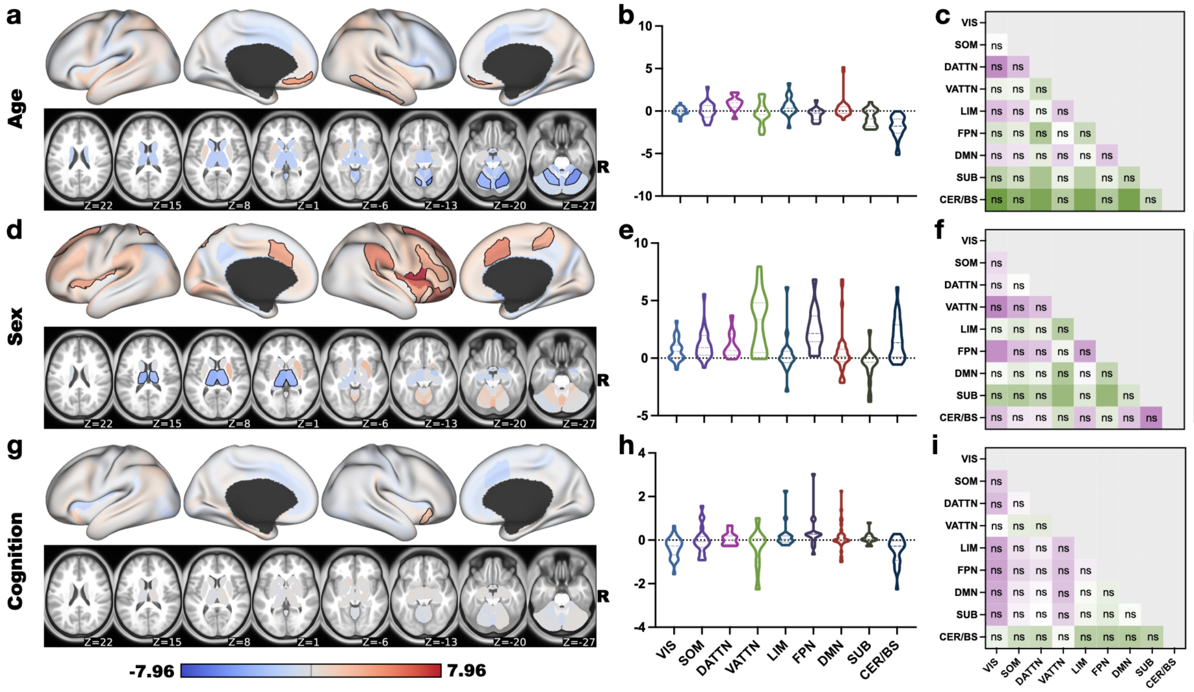

Supplementary Figure 7: Associations between SC-FC coupling (FS191) and age, sex, total cognition. **a d g** display regional β values from the GLM quantifying associations between SC-FC coupling and age, sex (blue indicates higher SC-FC coupling in females, red higher in males) and total cognition, respectively. Areas with significant β values (after correction) are outlined in black. **b e h** show the network-wise β values for age, sex and total cognition, respectively. **c f i** show the t-statistics for all pairwise comparisons of associations across networks for age, sex and cognition, calculated as the network on the y-axis versus the network on the x-axis. One-sided p values were calculated (see detailed description in the Methods section). Those comparisons with FDR corrected $p > 0.05$ are marked with ns.

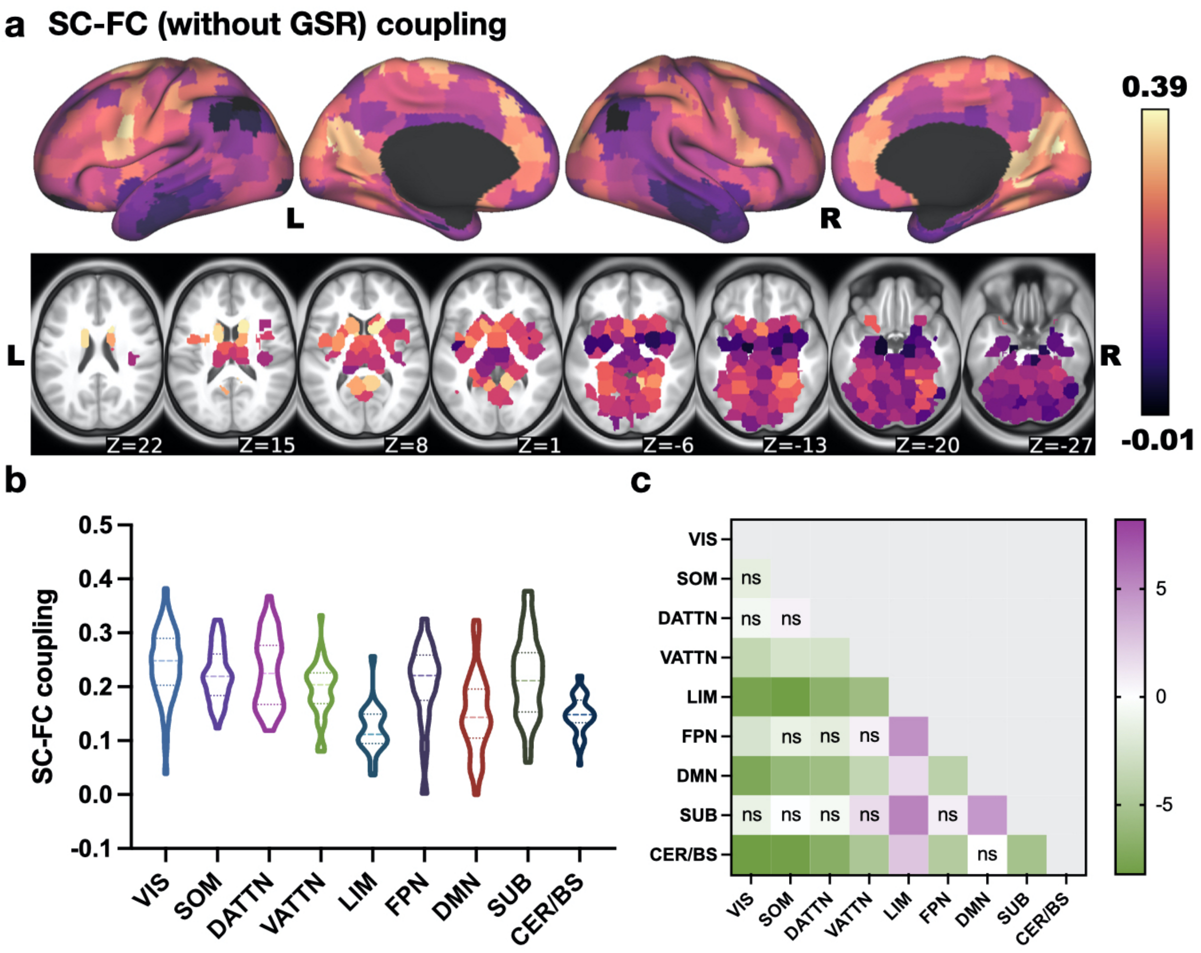

Supplementary Figure 8: SC-FC coupling computed using FC without global signal regression (GSR). **a** SC-FC (without GSR) coupling varies across cortical and subcortical areas with range from -0.01 to 0.39. **b** SC-FC (without GSR) coupling in nine networks. **c** shows the t-statistics for all pairwise comparisons of SC-FC coupling (no GSR) across networks, calculated as the network on the y-axis versus the network on the x-axis. One-sided p values were calculated (see detailed description in the Methods section). Those comparisons with FDR corrected $p > 0.05$ are marked with ns. Pearson's correlation of the SC-FC coupling results in the main paper (with GSR) with the non-GSR coupling was $r = 0.961$ (one-sided $p = 0$).

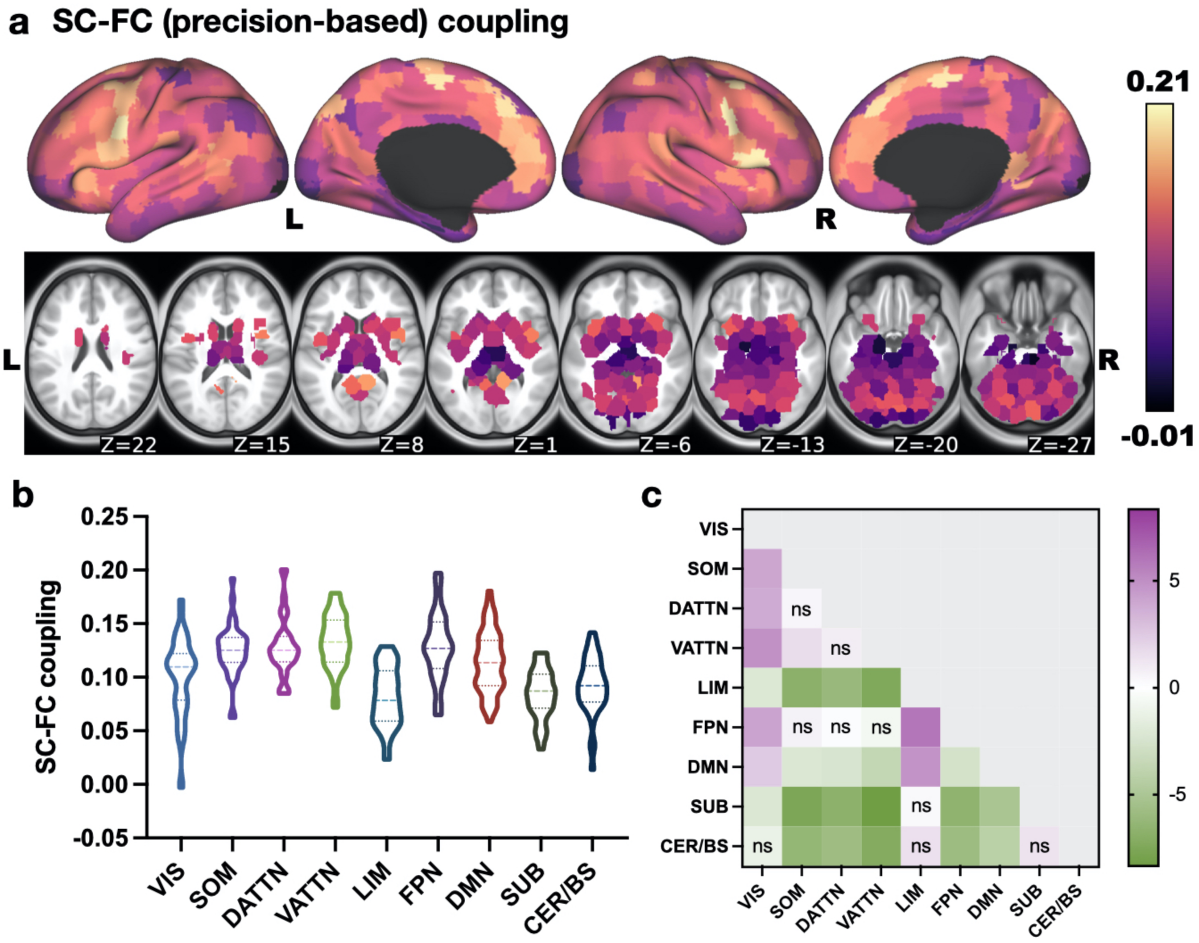

Supplementary Figure 9: SC-FC coupling calculated using precision-based FC. **a** SC-FC coupling across cortical, subcortical and cerebellar regions. **b** SC-FC coupling distribution across nine networks. Ventral/dorsal attention, frontal parietal and somatomotor networks had generally higher coupling than other areas. Limbic and subcortical area had weaker mean coupling (0.08 ± 0.03 and 0.08 ± 0.02, respectively). **c** shows the t-statistics for all pairwise comparisons of SC-FC coupling (precision-based) across networks, calculated as the network on the y-axis versus the network on the x-axis. One-sided p values were calculated (see detailed description in the Methods section). Those comparisons with FDR corrected $p > 0.05$ are marked with ns. Pearson's correlation of the SC-FC coupling results in the main paper (using full correlation-based FC) with the precision-based FC measure is $r = 0.486$ (one-sided $p = 0$).

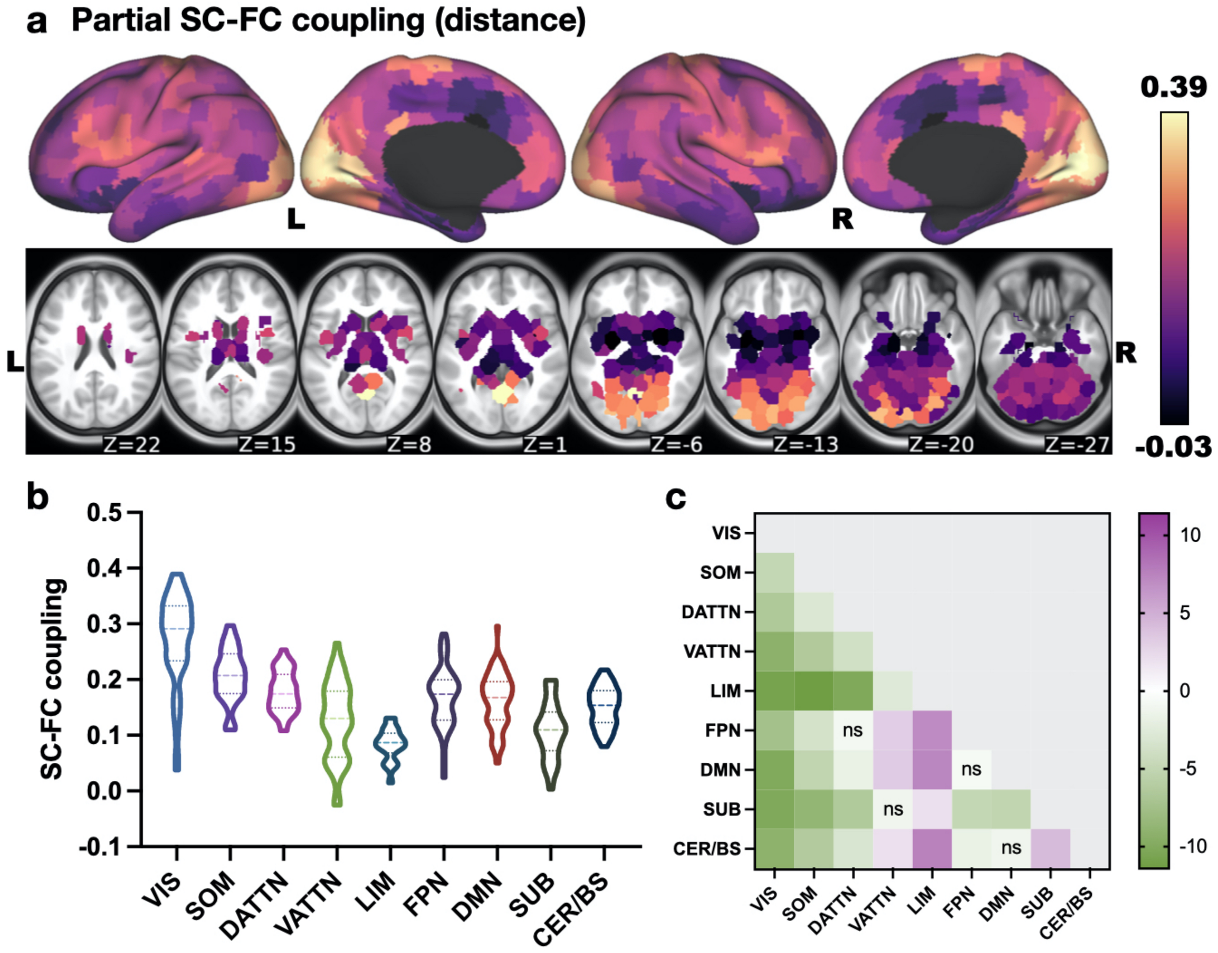

Supplementary Figure 10: Partial SC-FC coupling with inter-node Euclidean distance as a covariate. **a** Partial SC-FC coupling was computed by partial Spearman correlation of the row in SC and its corresponding row in FC with the Euclidean distance between regional centroid pairs as a covariate. SC-FC coupling measured in this way varies across cortical and subcortical areas and ranges from -0.03 to 0.39. **b** Partial SC-FC coupling in nine networks. **c** shows the t-statistics for all pairwise comparisons of partial SC-FC coupling across networks, calculated as the network on the y-axis versus the network on the x-axis. One-sided p values were calculated (see detailed description in the Methods section). Those comparisons with FDR corrected $p > 0.05$ are marked with ns. Visual and somatomotor network have significant higher partial SC-FC coupling than other networks. Limbic network has significantly weaker partial SC-FC coupling. Partial SC-FC coupling is correlated with the standard SC-FC coupling (Pearson's $r = 0.431$, one-sided $p = 0$).

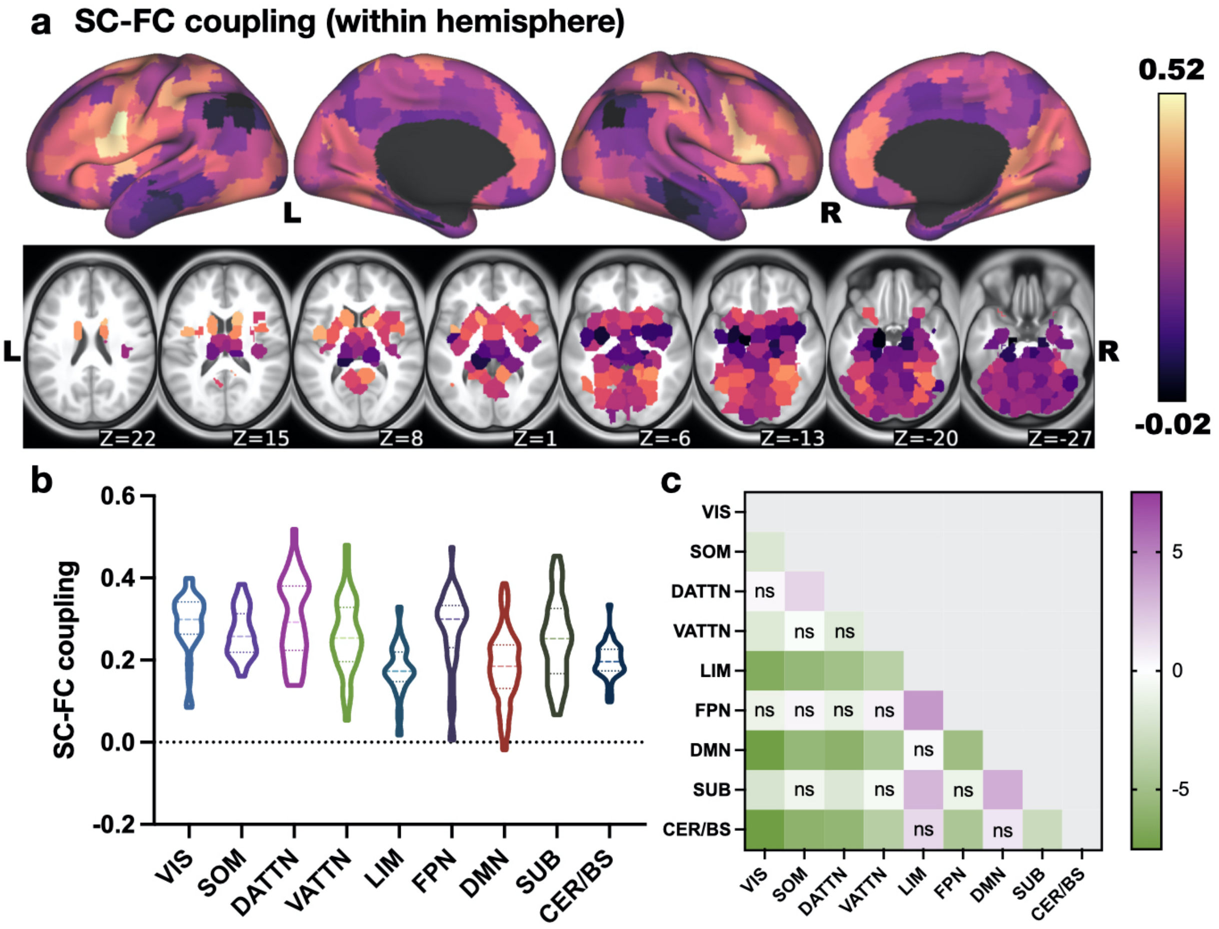

Supplementary Figure 11: SC-FC coupling within a single hemisphere. **a** SC-FC within hemisphere coupling varies across cortical and subcortical areas with range from -0.02 to 0.52, which is a bit higher than whole brain SC-FC coupling but preserves consistency with the whole-brain results. **b** SC-FC within hemisphere coupling in nine networks. **c** shows the t-statistics for all pairwise comparisons of SC-FC coupling within hemisphere across networks, calculated as the network on the y-axis versus the network on the x-axis. One-sided p values were calculated (see detailed description in the Methods section). Those comparisons with FDR corrected $p > 0.05$ are marked with ns. Pearson's correlation of the SC-FC coupling results in the main paper (using whole-brain SC/FC) with the single hemisphere SC/FC coupling is $r = 0.864$ (one-sided $p = 0$).

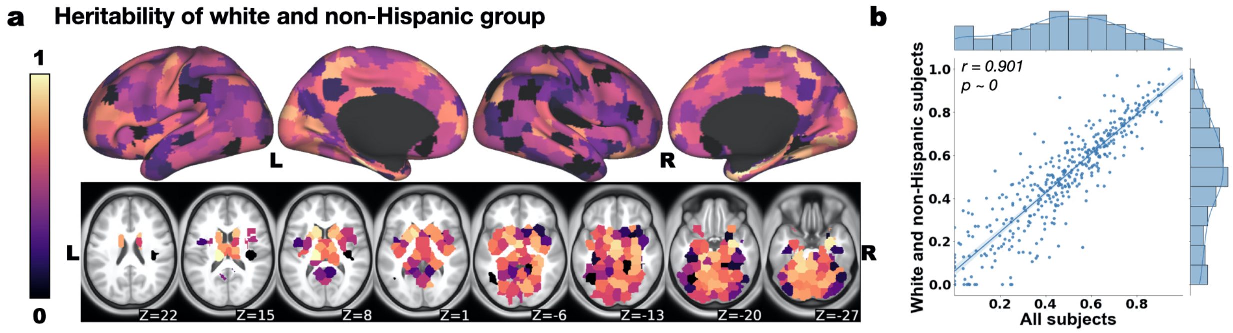

Supplementary Figure 12: Heritability of the homogenous sub-group: white and non-Hispanic group in HCP. **a** Heritability of white and non-Hispanic group ($n = 645$) ranges from 0 to 1. **b** The subgroup heritability is highly correlated (Pearson's $r = 0.901$, one-sided $p = 0$) with the heritability from all subjects presented in the main paper. The translucent bands around the regression line represent 95% confidence interval for the regression estimate.

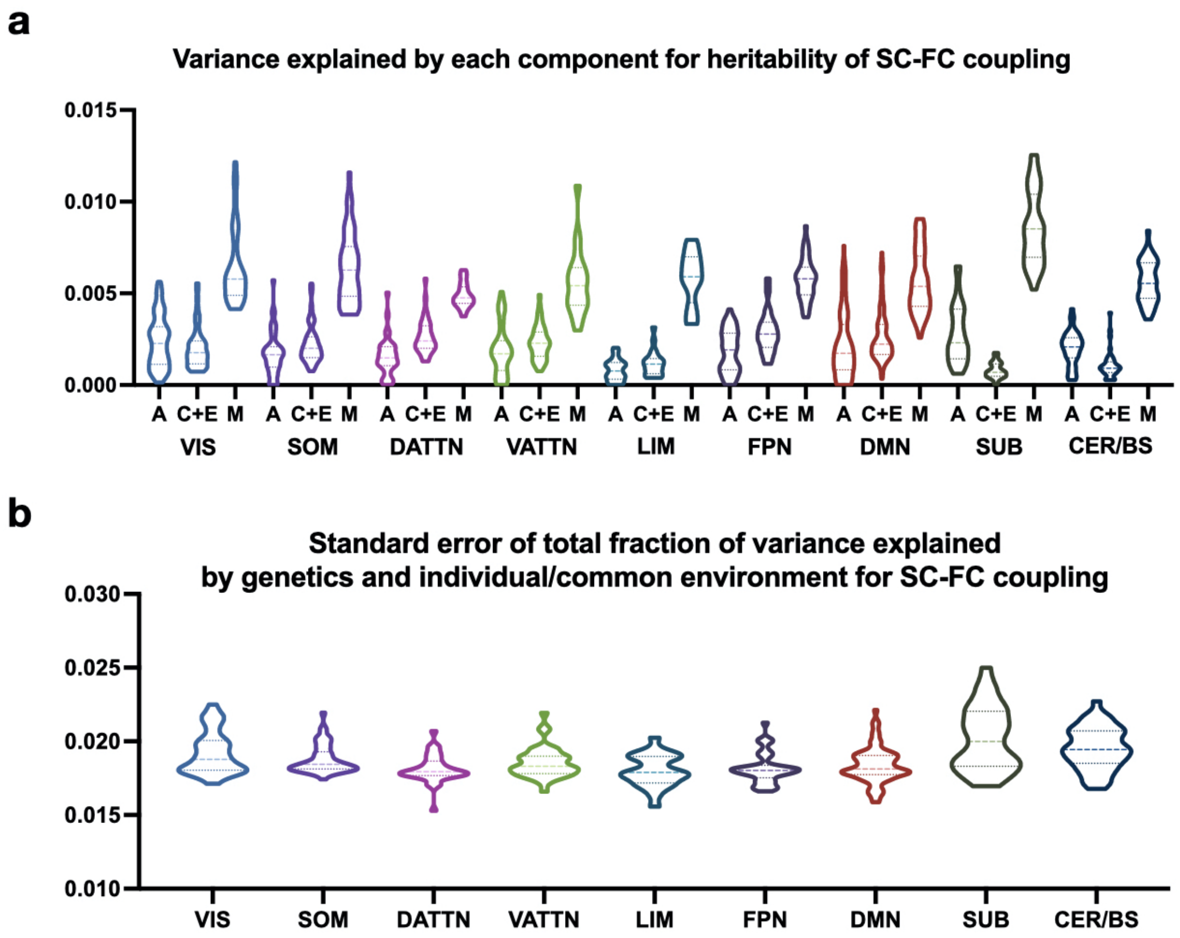

Supplementary Figure 13: **a** Variance explained by genetic effect (A), common environmental effect plus unique environmental effect (C+E), and intra-subject measurement error (M) for SC-FC coupling in 7 Yeo networks, subcortical regions and cerebellum/brain stem. **b** Standard error of total fraction of variance explained by all components ((A+C+E)/(A+C+E+M)) for SC-FC coupling in 7 Yeo networks, subcortical regions and cerebellum/brain stem.

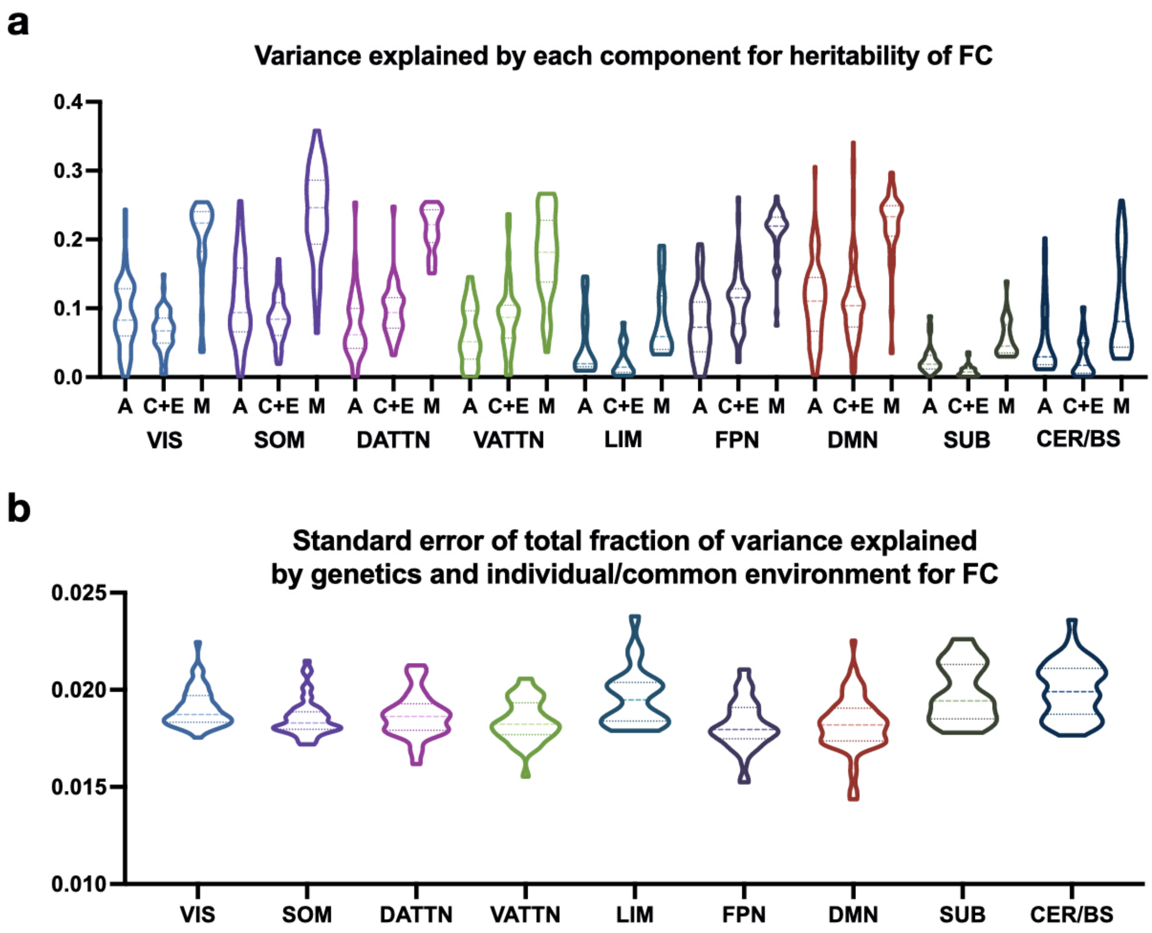

Supplementary Figure 14: **a** Variance explained by genetic effect (A), common environmental effect plus unique environmental effect (C+E), and intra-subject measurement error (M) for FC node strength in 7 Yeo networks, subcortical regions and cerebellum/brain stem. **b** Standard error of total fraction of variance explained by all components ((A+C+E)/(A+C+E+M)) for FC node strength in 7 Yeo networks, subcortical regions and cerebellum/brain stem.

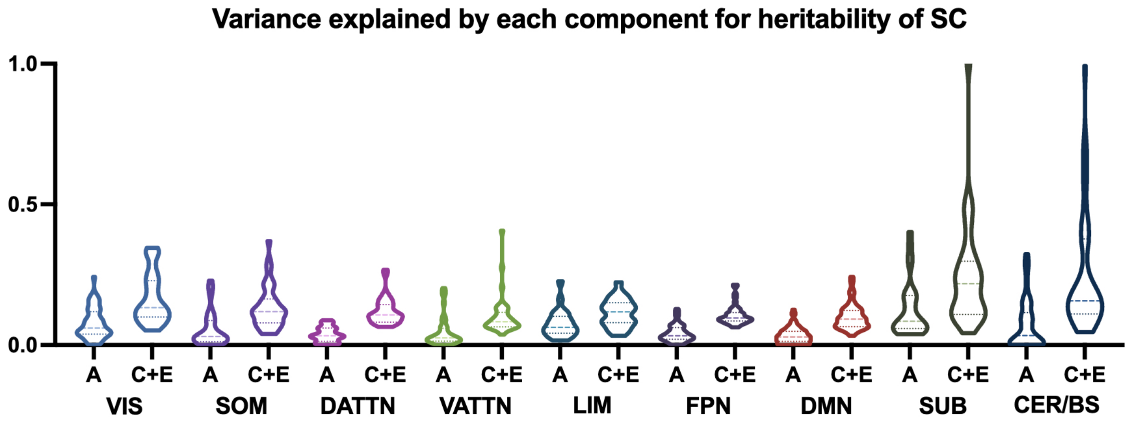

Supplementary Figure 15: Variance explained by genetic effect (A) and common environmental effect plus unique environmental effect (C+E) for SC node strength in 7 Yeo networks, subcortical regions and cerebellum/brain stem.
