# Supplementary material for: Heritability and interindividual variability of regional structure-function coupling
Source: Nat Commun. 2021 Aug 12;12:4894. doi: 10.1038/s41467-021-25184-4 (PMC8361191; doi:10.1038/s41467-021-25184-4)
Supplement: Supplementary file 3 — Reporting Summary [file 41467_2021_25184_MOESM3_ESM.pdf]

nature research

Corresponding author(s): Amy Kuceyeski

Last updated by author(s): Jul 2, 2021

Reporting Summary

Nature Research wishes to improve the reproducibility of the work that we publish. This form provides structure for consistency and transparency in reporting. For further information on Nature Research policies, see our Editorial Policies and the Editorial Policy Checklist.

Statistics

For all statistical analyses, confirm that the following items are present in the figure legend, table legend, main text, or Methods section.

n/a	Confirmed	
[ ]	[x]	The exact sample size (*n*) for each experimental group/condition, given as a discrete number and unit of measurement
[ ]	[x]	A statement on whether measurements were taken from distinct samples or whether the same sample was measured repeatedly
[ ]	[x]	The statistical test(s) used AND whether they are one- or two-sided<br>*Only common tests should be described solely by name; describe more complex techniques in the Methods section.*
[ ]	[x]	A description of all covariates tested
[ ]	[x]	A description of any assumptions or corrections, such as tests of normality and adjustment for multiple comparisons
[ ]	[x]	A full description of the statistical parameters including central tendency (e.g. means) or other basic estimates (e.g. regression coefficient) AND variation (e.g. standard deviation) or associated estimates of uncertainty (e.g. confidence intervals)
[ ]	[x]	For null hypothesis testing, the test statistic (e.g. *F*, *t*, *r*) with confidence intervals, effect sizes, degrees of freedom and *P* value noted<br>*Give P values as exact values whenever suitable.*
[x]	[ ]	For Bayesian analysis, information on the choice of priors and Markov chain Monte Carlo settings
[x]	[ ]	For hierarchical and complex designs, identification of the appropriate level for tests and full reporting of outcomes
[ ]	[x]	Estimates of effect sizes (e.g. Cohen's *d*, Pearson's *r*), indicating how they were calculated

Our web collection on statistics for biologists contains articles on many of the points above.

Software and code

Policy information about availability of computer code

Data collection	No software was used for data collection
Data analysis	Python code to reproduce the main results of this paper is publicly available at https://github.com/zijin-gu/scfc-coupling. The original code of the LME model for heritability estimation is publicly available at https://github.com/ThomasYeoLab/Standalone_Li2019_GSR/tree/master/external_packages/LME. The functional and diffusion data were already preprocessed by HCP. Example script to run HCP tractography can be found at https://github.com/zijin-gu/scfc-coupling/blob/main/example_mrtrix_script_cc400_ifod2act5Mfsl.sh. The denoising, ROI time series extraction, volume censoring and FC extraction code is publicly available at https://github.com/kjamison/fmriclean.

For manuscripts utilizing custom algorithms or software that are central to the research but not yet described in published literature, software must be made available to editors and reviewers. We strongly encourage code deposition in a community repository (e.g. GitHub). See the Nature Research guidelines for submitting code & software for further information.

Data

Policy information about availability of data

All manuscripts must include a data availability statement. This statement should provide the following information, where applicable:

- Accession codes, unique identifiers, or web links for publicly available datasets
- A list of figures that have associated raw data
- A description of any restrictions on data availability

HCP imaging and most behavioral data are publicly available at https://db.humanconnectome.org/ with the acceptance of HCP Open Access Data Use Terms. Some data elements, including family structure (e.g. whether a participant is a twin or a non-twin sibling), exact age, handedness, ethnicity and race, are available only to qualified investigators who agree to HCP's Restricted Data Use Terms. Applications for access to Restricted Data should be submitted by every investigator who will view and use the data, will be processed individually, and approval is on a case-by-case basis. The time to get the data is about two weeks. Once given the access,

researchers should abide by a prohibition against publishing certain types of individual data in combination that could make a person individually recognizable, or that could harm and embarrass someone who was inadvertently identified. The specific data used in this work is WU-Minn HCP Data - 1200 Subjects and WU-Minn HCP Retest Data. Source data to plot the figures are provided with this paper.

Field-specific reporting

Please select the one below that is the best fit for your research. If you are not sure, read the appropriate sections before making your selection.

[x] Life sciences [ ] Behavioural & social sciences [ ] Ecological, evolutionary & environmental sciences

For a reference copy of the document with all sections, see nature.com/documents/nr-reporting-summary-flat.pdf

Life sciences study design

All studies must disclose on these points even when the disclosure is negative.

Sample size	We did not collect the data, so no sample size calculation was performed. Our analysis included all 941 individuals from the HCP S1200 dataset that had all four resting-state scans and a diffusion MRI scan.
Data exclusions	We began by excluding subjects that did not have all 4 resting-state fMRI scans and a diffusion MRI scan. The heritability analysis (N = 941) included 116 MZ twin pairs, 61 DZ twin pairs, 455 full siblings and 132 singletons. For the SC-FC coupling analysis, we selected a random subset of 420/941 subjects that were unrelated. For the age, sex and cognition association analysis, we used 415/420 unrelated individuals as 5 subjects did not have cognitive scores.
Replication	There were a subset of 41 subjects in the HCP dataset that had a second scan 6 months later; Bland-Altman plots and test-retest correlations revealed high level of consistency in the SC-FC coupling measure across time within the same individual. In addition, we performed out-of-sample replication of SC-FC coupling values by taking an independent set of 346 unrelated individuals and showing high correlations with the original SC-FC coupling results on the 420 unrelated individuals.
Randomization	We did not randomize any participants into different experimental groups. To do the individual variability analyses, unrelated subjects were needed. To do the heritability analyses, the subjects with family information were needed.
Blinding	Blinding is not relevant to this study. We need to know the subjects' relateness information to select them to do certain analysis.

Reporting for specific materials, systems and methods

We require information from authors about some types of materials, experimental systems and methods used in many studies. Here, indicate whether each material, system or method listed is relevant to your study. If you are not sure if a list item applies to your research, read the appropriate section before selecting a response.

Materials & experimental systems

n/a	Involved in the study	
[x]	[ ]	Antibodies
[x]	[ ]	Eukaryotic cell lines
[x]	[ ]	Palaeontology and archaeology
[x]	[ ]	Animals and other organisms
[x]	[ ]	Human research participants
[x]	[ ]	Clinical data
[x]	[ ]	Dual use research of concern

Methods

n/a	Involved in the study	
[x]	[ ]	ChIP-seq
[x]	[ ]	Flow cytometry
[ ]	[x]	MRI-based neuroimaging

Magnetic resonance imaging

Experimental design

Design type	Resting-state functional MRI
Design specifications	Two 15 minute scans per session, and two sessions over two consecutive days. A subset of subjects had a second set of scans approximately 6 months later with the same protocol
Behavioral performance measures	No behavioral measures were taken during the scan.

Acquisition

Imaging type(s): resting-state MRI, diffusion and anatomical MRI

Field strength: 3T

Sequence & imaging parameters: Four gradient-echo EPI resting-state fMRI runs (2.0mm isotropic voxels, TR/TE = 720/33.1ms, 8x multiband acceleration, FoV = 208 x 180 mm^2, Flip Angle = 52 degrees, 72 slices) of approximately 15 minutes each, with two runs in one session and two in a second session, where each session included both right-left and left-right phase encoding.

T1-weighted anatomical scans were acquired using a 3D MPRAGE sequence with 0.7mm isotropic resolution (FOV=224 x 224mm^2, matrix=320, 256 sagittal slices, TR=2400ms, TE=2.14ms, TI=1000ms, FA=8°, GRAPPA = 2). T2-weighted anatomical scans were acquired using a variable flip angle turbo spin-echo sequence (Siemens SPACE) with 0.7 mm isotropic resolution with the same FOV and slices as the MPRAGE (TR = 3200 ms, TE = 565 ms, GRAPPA = 2)

Area of acquisition: whole brain field of view

Diffusion MRI: [x] Used [ ] Not used

Parameters: 1.25mm isotropic voxels, TR/TE =5520/89.5ms, 3x multiband acceleration, b=1000,2000,3000, 90 directions/shell, collected with both left-right and right-left phase encoding.

A multi-shell, multi-tissue constrained spherical deconvolution (CSD) model was computed in MRtrix3 to estimate the orientation distribution function (Jeurissen 2014). We used a probabilistic (iFOD2, Tournier 2010), anatomically constrained (ACT, Smith 2012) tractography algorithm with dynamic seeding to create individual, whole-brain tractograms containing 5 million streamlines. To better match the whole brain tractogram to diffusion properties of the observed data, we also computed streamline weights that are designed to reduce known biases in tractography data (SIFT2, Smith 2015). Finally, the tractograms were used to estimate SC weights for the CC400 (Craddock 2012) atlas. The SC between any two regions was the SIFT2-weighted sum of streamlines connecting those regions divided by the sum of the gray matter volume of those regions. The result was an ROI-volume normalized pairwise SC matrix for each subject. An example script to run HCP tractography can be found at https://github.com/zijin-gu/scfc-coupling/blob/main/example_mrtrix_script_cc400_ifod2act5Mfsl.sh.

Preprocessing

Preprocessing software: We used minimally processed data from the HCP S1200 release, which were preprocessed by the HCP consortium using the Minimal Preprocessing Pipeline (MPP, Glasser 2013) that combines tools from FSL 5.0.9, FreeSurfer 5.3, and Connectome Workbench. Smoothing was not applied to the data used in our study. The denoising, ROI time series extraction, volume censoring and FC extraction code is publicly available at https://github.com/kjamison/fmriclean.

Normalization: Volumetric normalization was performed as part of the MPP using FSL FNIRT

Normalization template: HCP data have been normalized to the MNI152 v6 asym template

Noise and artifact removal: HCP-provided data have been denoised using a very liberal high-pass filter (0.0005 Hz), motion parameter regression, and ICA-FIX (Salimi-Khorshidi, 2014) to automatically reject components classified as structured noise based on spatial and temporal features. We additionally regressed the global gray-matter signal, its temporal derivative, and any remaining linear trend.

Volume censoring: We identified outlier volumes where global signal or its temporal derivative exceeded 5 std from the mean, or where framewise displacement exceeded 0.9mm. The first 10 volumes of each scan were also automatically discarded. Outlier volumes were ignored during global-signal regression, and during computation of covariance matrices.

Statistical modeling & inference

Model type and settings: We used a Generalized Linear Model, which is a mass univariate approach, to measure the association between regional SC-FC coupling and age, sex and cognition. We included covariates of years of education, intracranial volume (ICV), in-scanner head motion as well as the two-way interactions terms of age*total cognition score, sex*total cognition score, education*total cognition score and ICV*motion in the model.

For the heritability analysis, we used the linear mixed effects model proposed in Ge et al. (2017). This approach was developed to quantify heritability of functional connectome fingerprints with respect to the inter-subject component, while removing the effect of transient changes across observations of a single subject. This approach allows examination of the association between the genetic relationship and phenotypic similarity, while accounting for shared environment of siblings. Age, sex and handedness were used as fixed-effects covariates.

Effect(s) tested: This was a resting-state scan, so no effects were tested.

Specify type of analysis: [ ] Whole brain [x] ROI-based [ ] Both

Anatomical location(s): The CC400 (Craddock, 2012) atlas is defined in MNI template volume space and includes 392 cortical and subcortical parcels of relatively uniform size. The atlas was masked by each subject's FreeSurfer-derived gray-matter mask before using it to parcellate functional or tractography data.

Statistic type for inference
(See Eklund et al. 2016)

We did not use voxel-wise or cluster-wise statistics

Correction

We used false discovery rate correction to adjust for multiple comparisons over the 392 regions in the CC400 atlas

Models & analysis

n/a	Involved in the study
☐	☒ Functional and/or effective connectivity
☒	☐ Graph analysis
☐	☒ Multivariate modeling or predictive analysis

Functional and/or effective connectivity

For each subject, functional connectivity between ROI pairs was estimated from the concatenated, z-normalized time series using either Pearson correlation between time series or Tikhonov-regularized precision matrices. A single Tikhonov regularization parameter of 0.3 was selected to maximize similarity between regularized subject precision matrices and the group averaged unregularized precision matrix.

Multivariate modeling and predictive analysis

We used a Generalized Linear Model, which is a mass univariate approach, to measure the association between regional SC-FC coupling and age, sex and cognition. We included covariates of years of education, intracranial volume (ICV), in-scanner head motion as well as the two-way interactions terms of age*total cognition score, sex*total cognition score, education*total cognition score and ICV*motion in the model.

For the heritability analysis, we used the linear mixed effects model proposed in Ge et al. (2017). This approach was developed to quantify heritability of functional connectome fingerprints with respect to the inter-subject component, while removing the effect of transient changes across observations of a single subject. This approach allows examination of the association between the genetic relationship and phenotypic similarity, while accounting for shared environment of siblings. Age, sex and handedness were used as fixed-effects covariates.
